# Summation of connectivity strengths in the visual cortex reveals stability of neuronal microcircuits after plasticity

**DOI:** 10.1186/s12868-015-0203-1

**Published:** 2015-10-09

**Authors:** Lyes Bachatene, Vishal Bharmauria, Sarah Cattan, Nayan Chanauria, Jean Rouat, Stéphane Molotchnikoff

**Affiliations:** Laboratoire de Neurosciences de la vision, Département de Sciences Biologiques, Université de Montréal, CP 6128 Succ. Centre-Ville, Montréal, QC H3C 3J7 Canada; Neurosciences Computationnelles et Traitement Intelligent des Signaux (NECOTIS), Département de Génie Électrique et Génie Informatique, Université de Sherbrooke, Sherbrooke, QC Canada

**Keywords:** Visual cortex, Plasticity, Summation, Correlation, Adaptation

## Abstract

**Background:**

Within sensory systems, neurons are continuously affected by environmental stimulation. Recently, we showed that, on cell-pair basis, visual adaptation modulates the connectivity strength between similarly tuned neurons to orientation and we suggested that, on a larger scale, the connectivity strength between neurons forming sub-networks could be maintained after adaptation-induced-plasticity. In the present paper, based on the summation of the connectivity strengths, we sought to examine how, within cell-assemblies, functional connectivity is regulated during an exposure-based adaptation.

**Results:**

Using intrinsic optical imaging combined with electrophysiological recordings following the reconfiguration of the maps of the primary visual cortex by long stimulus exposure, we found that within functionally connected cells, the summed connectivity strengths remain almost equal although connections among individual pairs are modified. Neuronal selectivity appears to be strongly associated with neuronal connectivity in a “homeodynamic” manner which maintains the stability of cortical functional relationships after experience-dependent plasticity.

**Conclusions:**

Our results support the “homeostatic plasticity concept” giving new perspectives on how the summation in visual cortex leads to the stability within labile neuronal ensembles, depending on the newly acquired properties by neurons.

## Background

Visual processing in the brain highly depends on physiological connectivity of neurons to establish functional circuits in the visual cortex. Specific neuronal connections are framed between stimulus selective neurons (functional circuits) within cell-assemblies that process visual information [1]. These recruited functional circuits, when co-activated, encode the attributes of stimuli [2] and are believed to be crucial for visual perception [1]. It is well established that neurons sharing similar selectivity vigorously and strongly connect with each other in response to the visual stimulation [1, 3, 4].

In a recent report [5], we showed that neurons exhibit changes in the correlation-strength after adaptation for their original optimal and new acquired optimal orientations suggesting that adaptation impacts the strength of their functional connections. These previous data were investigated on cell-pairs basis and focused exclusively on cells sharing similar orientations before and after adaptation phase. Hence the previous report was not centered on the large spectrum of orientations. In the present paper, seeking a deeper understanding how connectivity-strength is modified, we further broadened analyses by investigating the connection strengths between cells selective to a wide range of orientations as revealed within a cluster of neurons. Thus, crosscorrelograms were computed between all cells of a cluster irrespective of the axis of the preferred orientation. The magnitudes of the central pic were computed to derive the strength of inter-neuronal functional relationships and then we investigated the modulation of crosscorrelogram pics following adaptation. In addition, the present paper focuses on a summative model which explains how within cell-assemblies formed by similarly tuned and differently tuned neurons, the summed connectivity strength remained relatively unchanged during plasticity. We recorded visual responses from neuronal units and populations using extracellular electrophysiological recordings and intrinsic optical imaging. Brain plasticity is an inherent feature of cortical neurons that is inevitable for animals to adapt to the environment. The cortical organization is well known to be malleable mostly during early stages of life [6, 7]. For instance, visual neurons of animals raised in a forced, stripped environment exhibit orientation-preference shifts toward the imposed stimulus [7]. Such plastic changes have been widely observed at neuronal [8–11] and populational levels [10, 12, 13]. In principle, these changes are attributed to visual deprivation or visual training (adaptation).

Crosscorrelations have been widely employed to reveal the putative functional connections between neurons [14–16]. We crosscorrelated the spiking activity of simultaneously recorded neurons to reveal the functional relationships between them.

Our results are in line with the concept of homeostatic plasticity [17–19]. Indeed, a homeostatic process is established in order to stabilize the initial global connectivity strength of the neuronal group [19]. This regulatory activity is considered as a complementary process to the Hebbian plasticity wherein changes of synaptic strength are observed in order to redefine the properties of neuronal-assemblies [19–21].

## Methods

### Ethical approval

Animal surgery procedures and electrophysiological recordings followed the guidelines of the Canadian Council on Animal Care and were approved by the Institutional Animal Care and Use Committee of the University of Montreal. Animals were supplied by the Division of Animal Resources of the University of Montreal. The experiments were conducted in accordance with the Guide for Care and Use of Laboratory Animals of the National Institutes of Health (USA).

### Animal surgery

Briefly, electrophysiological recordings and optical imaging were performed within layer II/III of V1 area of adult anaesthetized cats (Felis catus). Eight adult cats (2.5–3.5 kg, age 12–24 months) of either sex were used for this study. General anaesthesia was maintained by artificial ventilation with a mixture of N_2_O/O_2_ (70:30) supplemented with 0.5 % isoflurane (AErrane, Baxter, Toronto, ON, Canada) for the duration of the experiment. The following parameters were monitored throughout the experiment: the EEG, the expired CO_2_, the temperature and the heart rate. At the end of each experiment, euthanasia was achieved with a lethal dose of pentobarbital sodium (Somnotol, MTC Pharmaceuticals, Cambridge, ON, Canada; 100 mg kg^−1^) by intravenous injection. Details are described in Bachatene et al. [5].

### Electrophysiology

Visual stimuli were generated with a VSG 2/5 graphic board (Cambridge Research Systems, Rochester, England) and displayed on a 21-in. monitor (Sony GDM-F520 Trinitron, Tokyo, Japan) placed 57 cm from the cat’s eyes, with 1024 × 768 pixels, running at 100 Hz frame refresh. Stimuli were drifting sine-wave grating square patches (~2°–5°) covering the excitatory RF (unidirectional movement). Patches characteristics were set to evoke optimal responses: contrast at 80 %, mean luminance at 40 cd/m^2^, optimal spatial and temporal frequencies set within the 0.1–0.5 cycles/deg. and 1.0–2.0 Hz range, respectively. In all cases the above parameters were chosen with the aim of evoking the maximal discharges. After manual RF characterization, nine oriented stimuli centered on the preferred orientation were selected and used for the rest of the experiment. Test orientations were applied in random order. Each oriented stimulus was presented in blocks of 25 trials lasting 4.1 s each, with a random inter-trial interval (1.0–3.0 s) during which no stimuli were presented. Thus, a recording session lasted for 25–30 min. Peri-stimulus time histograms were recorded. Once control orientation tuning curves were characterized, an adapting non-preferred stimulus was presented continuously for 3 or 12 min and 24 min in one experiment. The adapting stimulus was a drifting grating whose orientation was randomly selected in the range 22.5°–67.5° off of the neuron’s preferred orientation. All other stimulus parameters were kept constant, at control values, throughout the recordings. Neurons were isolated from multi-unit activity using autocorrelograms, principal component analysis, spike wave-shapes and cluster separation. Details are described in Bachatene et al. [5].

### Optical imaging

Detailed account of intrinsic optical imaging is available in Cattan et al. [12]. Intrinsic optical imaging allows assessment of the activity of a large population of cells. Thus, we used this technique to visualize the range of shift propagation following adaptation.

After craniotomy, the dura mater was removed, a round chamber (15 mm in diameter) was fixed with dental cement above one hemisphere’s area 17 and the chamber was filled with mineral oil and closed with a cover glass.

Achromatic gratings were to stimulate all cortical area within the imaging window to obtain orientation maps in control and post-adaptation sessions (contrast: 75 %; generated by VSG software; Cambridge Research Systems, Rochester, UK), presented randomly in order to avoid stimulus-order bias with rotation in eight different orientations from 0° to 157.5°, spatial frequency: 0.3 cycles/°, temporal frequency: 1 Hz). Each trial started with the presentation of a black screen for 15 s, and this was followed by the presentation of every orientation (12 s). For each presented orientation spanning 12 s, the grating was kept stationary during the first 6 s to remove the cortical activity resulting from the stimulus onset, and this was followed by drifts in one direction for the next 3 s and then in the reverse direction for 3 s to maximize cortical responses. The stimulation was full-screen. From these recordings, we generated control polar orientation maps. Then we presented a patch as an adapter for 12 min (full screen stimulus). Immediately after adaptation, we stimulated again all cortical area within the imaging window by presenting full screen stimuli as in the control phase. The polar map obtained post-adaptation was compared with the control map to evaluate the spatial spread of local adaptation.

### Data acquisition and processing

The cortex was illuminated with 630-nm light. Cortex images were captured with a CCD camera (Dalsa 1 M60P; Dalsa, Waterloo, Ontario, Canada), composed of two 50-mm f1.2 lenses arranged in tandem, and focused 500 µm below the cortical surface. Images were digitised with Imager 3001 (Optical Imaging, Germantown, NY, USA), with a spatial resolution of 1024 × 1024 pixels (binned 2 × 2), and a temporal resolution of 20-ms frame duration. Image analysis was performed with MATLAB programs (MathWorks, Natick, MA, USA).

Thirty images were recorded for every orientation. As the last images showed more activity than the initial images, the average of the last 10 images (21–30) was divided by the average of the first 20 images (1–20). This calculation was performed to remove the non-specific activity in initial images, while preserving the specific activity recorded mostly in the last frames. Then, the generalised indicator function method [22] was applied. In short, this method extracts the frames that account for as much of the signal as possible by using principal component analysis, and optimises the differences between signal and noise.

### Pixel shifts

To quantify changes in the orientation of pixels between control and post-adaptation polar maps, the amplitude of the shift in orientation was calculated from pairs of pixels located at the same position in the two maps.

$$ s_{i,j} = { \hbox{min} }\left( {\left| {p2_{i,j} - p1_{i,j} } \right|,180 - \left| {p2_{i,j} - p1_{i,j} } \right|} \right) $$

where s is the shift-amplitude associated with the pair of pixels at the map position (i, j); p1 the pixel-orientation in the first map and, p2 the orientation of the same pixel in the second map. The shift-map comprised of all the shifts calculated for each position.

### Connectivity strength, crosscorrelograms and shift predictor

Crosscorrelograms (CCG’s) were performed in order to compare the spike distribution of each neuron of the pair within a time-frame; one neuron is set as reference and the second as target; this allows us to show the firing of the target neuron at a specific time-spread in relation to the firing of the reference neuron.

Time axis is divided into bins. The first bin is defined as: XMin, XMin + Bin. The next bin is XMin + Bin, XMin + Bin*2, etc. We calculated the distances from each spike to all spikes of the spike train as follows:

$$ d\left[ i \right] = ts\left[ i \right] - ref\left[ k \right] $$

where ts[i] represents the spike train, and ref[k] is each timestamp.

Bin counts were then divided by the number of reference events to normalize the counts per bin into probabilities. 95 % statistical threshold for the significance of the bins was used. Each bin-width was set at 1 ms. The connectivity strengths were calculated from the counts/bin as follows:

$$ CS = F \times b $$

where F is the neuron frequency and b represents the bin size of the calculated firing of the neuron.

The neuron frequency F was calculated as follows:

$$ F = \frac{N}{T} $$

where T represents the total time interval and N the number of spikes within this interval.

The 95 % confidence limit was calculated assuming that the expected bin count (EBC) has a Poisson distribution:

$$ EBC = CS \times Nref $$

where Nref is the number of reference events.

The 95 % confidence limit is calculated as follows:

$$ Low\,Conf. = x \,such\, that \,Prob \left( {S < x} \right) = 0.005 $$

$$ High\, Conf. = y\, such \,that \,Prob \left( {S > y} \right) = 0.005 $$

where S represents a random variable which has a Poisson distribution with parameter EBC.

Raw CCG’s were corrected by subtracting a shift-predictor algorithm in order to eliminate the putative significant peaks due to the simultaneous stimulation of both cells during each trial.

## Results

**Fig. 1:**
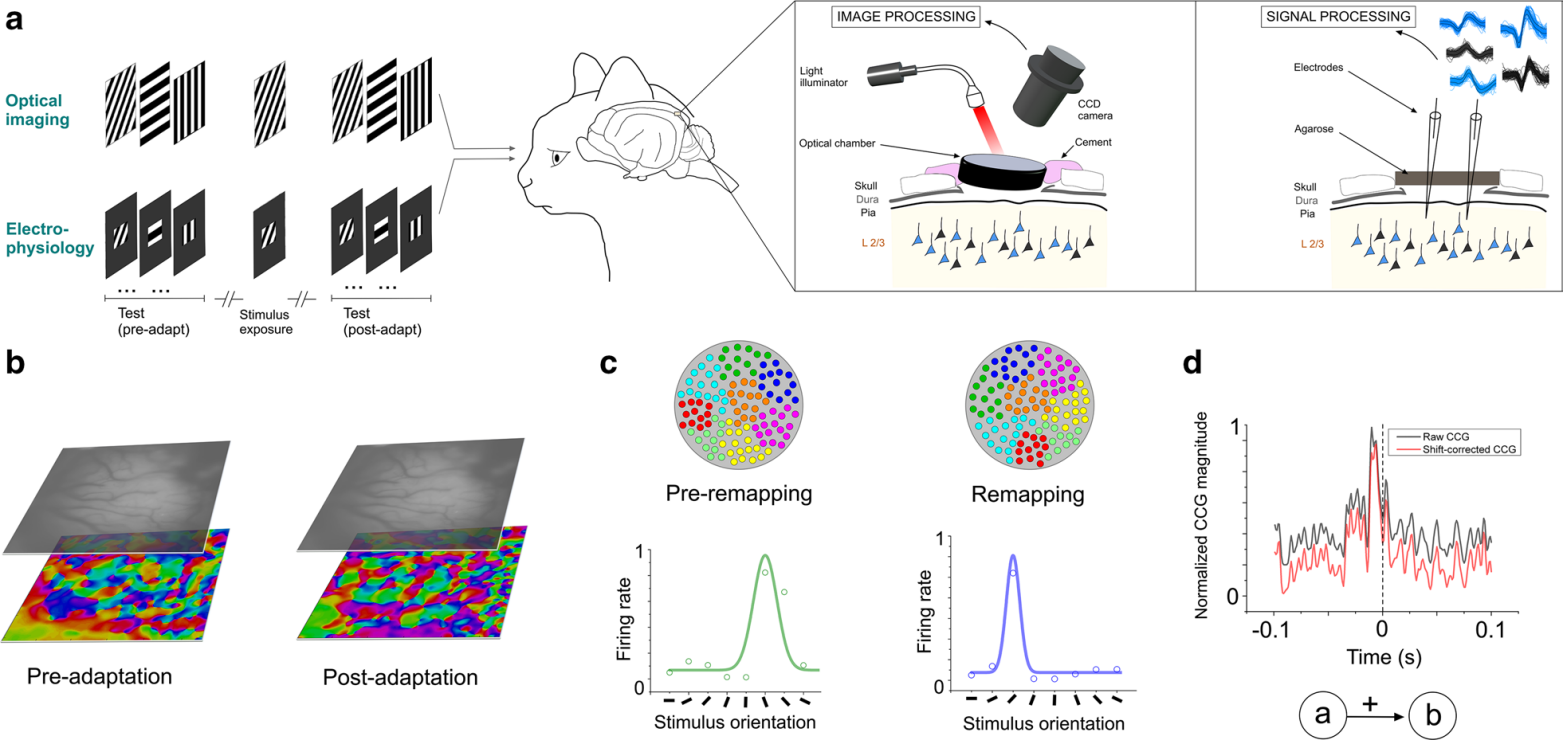
Neuronal recordings, orientation selectivity and crosscorrelation. **a** Visual stimulations, oriented gratings are presented randomly; one non-preferred grating is used for stimulus-exposure as adaptation (duration: 12 min). Visual responses are measured using intrinsic optical imaging and electrophysiological recordings. **b** Examples of orientation maps pre- and post-adaptation. **c** Model of plasticity of orientation maps and tuning curves shifts of one neuron (simulated data). **d** Raw (*gray*) and corrected (*red*) crosscorrelograms showing a mono-projection from neuron “a” to neuron “b”

To investigate the temporal relationships of spikes between cells of recorded assemblies, we performed optical imaging and extracellular electrophysiological recordings in V1 of adult anaesthetized cats based on an exposure-learning procedure (Fig. 1a). Optical imaging allows examining orientation shifts of a population of neurons [12] whereas electrophysiology permits recording a small number of neurons. Orientation preference maps were generated in order to compare the orientation layout before and after stimulus exposure: 3 or 12 min presentation of one particular orientation. An example of orientation maps is shown in Fig. 1b for both conditions (pre and post-adaptation periods). In addition to the optical imaging, electrophysiological recordings reveal, at the single cell scale, the orientation preference of cells and the changes of their selectivity after adaptation (orientation maps and neuronal tuning curves are shown in Fig. 1c, simulated data [23]). Colored dots within the circles depict orientation domains before (left) and following adaptation (right). Two orientation tuning curves are illustrated. Crosscorrelogram (CCG) analysis was used to unveil the putative inter-neuronal functional connections between neurons within a cell-assembly [15]. Raw CCGs were shift-corrected to eliminate the putative significant peaks due to the simultaneous stimulation of both cells (Fig. 1d). In this example, the CCG shows a significant peak within 10 ms time-window before zero mark. This suggests that cell A functionally projects onto cell B (cell B being the reference cell and cell A was set as a target neuron).

### Stability of pixel proportion in orientation domains

In the following section, we examined the orientation selectivity on a populational level using intrinsic brain imaging. For this purpose, area 17 was probed in order to perform computations of pixel-changes of orientation and pixel-distribution in the region of interest.

**Fig. 2:**
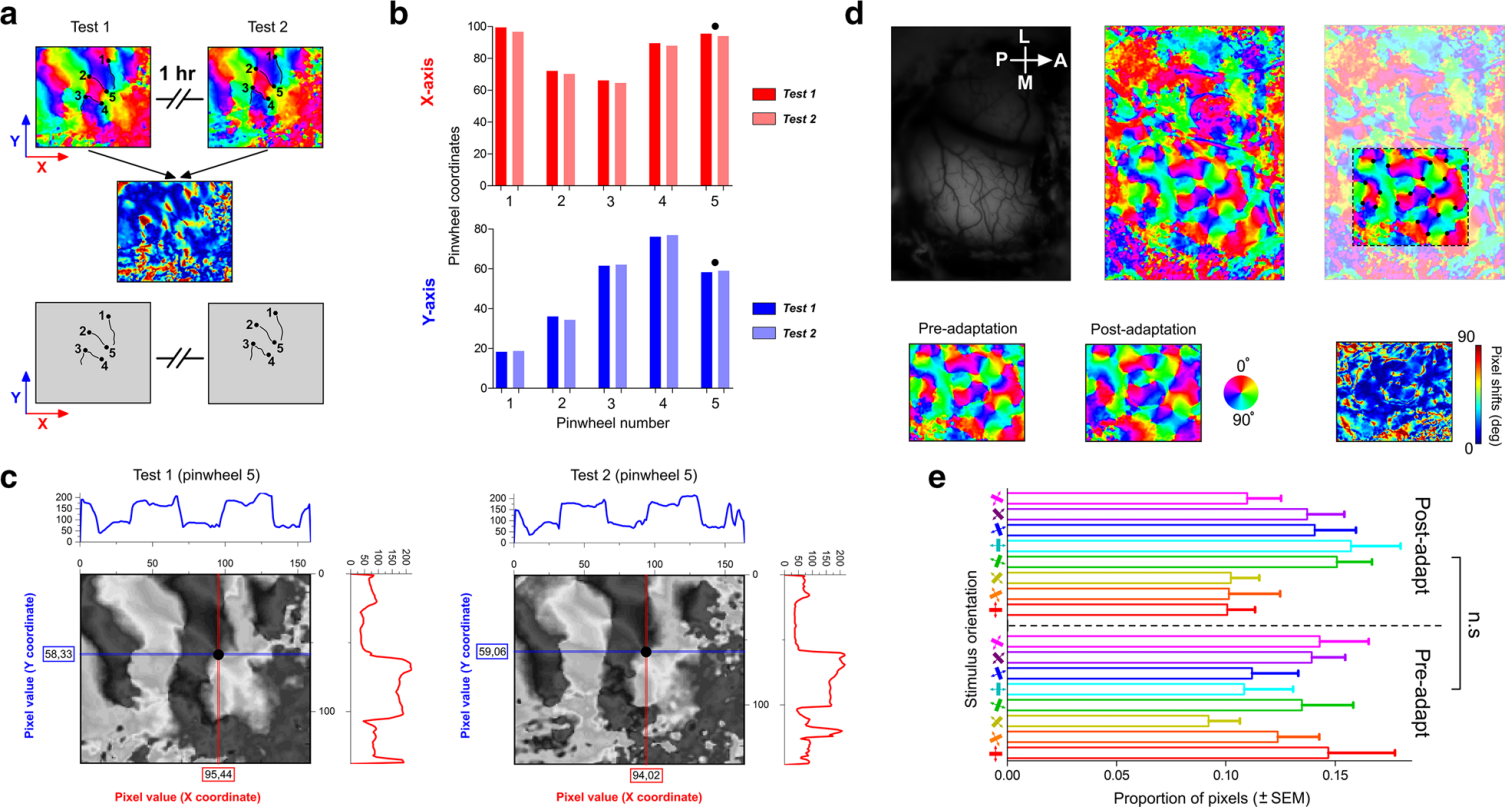
Orientation maps stability and Pixel proportions in orientation maps. **a** Two orientation maps generated at two intervals (1 h). Pinwheels and zones separating iso-orientation domains were traced within each map. The shift map in illustrated below both orientation maps (test 1 and test 2). **b** Spatial coordinates (*x* and *y axes*) of each pinwheel during both tests. The *black dot* indicates the pinwheel presented in (**c**). **c** Profile plot of pinwheel 5 with its respective coordinates for both tests. **d** Orientation maps pre- and post-adaptation, each color represents one specific orientation. The *dashed rectangle* indicates the region of interest wherein each *black dot* represents a pinwheel. Pixel-shifts map generated using pixel-by-pixel subtraction between the two maps (pre- and post-adaptation) and is shown on the *lower right part*. **e** Pixel proportions of all orientations for both pre- and post-adaptation conditions

To attribute the observed shifts in orientation maps to the effect of adaptation, we performed control tests of the stability of the maps over a period of time (1 h). From these maps, pinwheel spots and regions between two iso-orientation domains were identified and traced in both maps. An example of two generated maps is illustrated in Fig. 2a. A shift map was generated between test 1 and test 2. We observed small shifts in the frontiers between iso-orientation domains. This may be attributed to the small displacement of the pixels which may result from cortical movement, animal breathing…etc. However, in the shift map between pre- and post-adaptation, the shifts are more likely to happen in several regions of the map (see the description of Fig. 2d below).

As a further control of the stability of both maps, Pearson coefficient was computed between test 1 and test 2. We found a Pearson coefficient of 0.49 which is equivalent to a map similarity-index of 0.7 as previously shown [13, 43–45].

The spatial coordinates of each pinwheel was computed to test the stability of both orientation maps as shown in Fig. 2b for X-axis (red) and Y-axis (blue). The black dot represents the example shown in Fig. 2c (pinwheel 5). The similarity of both profiles is suggestive of the stability of the maps and thus the changes may be related to adaptation effect.

Following 12 min of stimulus exposure to one particular oriented grating (generally 90°), we observed a rearrangement of the orientation map characterized by pixel-shifts after adaptation. Figure 2d illustrates an orientation map generated from intrinsic optical imaging computations. In this investigation, we focused on the distribution of pixels for each orientation inside the region of interest for control and post-adaptation maps. This region of interest was selected based on pinwheel organization which is common to species such as cats and monkeys [24, 25].

To assess the magnitude of orientation shifts after visual adaptation, pixels were subtracted between the control and post adaptation steps. The result of this subtraction identified as pixel-shifts map is displayed in Fig. 2d (lower right); the color-scale represents the shift-intensity.

It is interesting to highlight that the global proportion of pixels is maintained, in other words, a new cortical map emerges following adaptation with a new distribution of pixels for each orientation, yet the proportion remained unchanged (One-way Anova test between pre- and post-adaptation, p > 0.05, Fig. 2e).

### Functional connectivity modulation

**Fig. 3:**
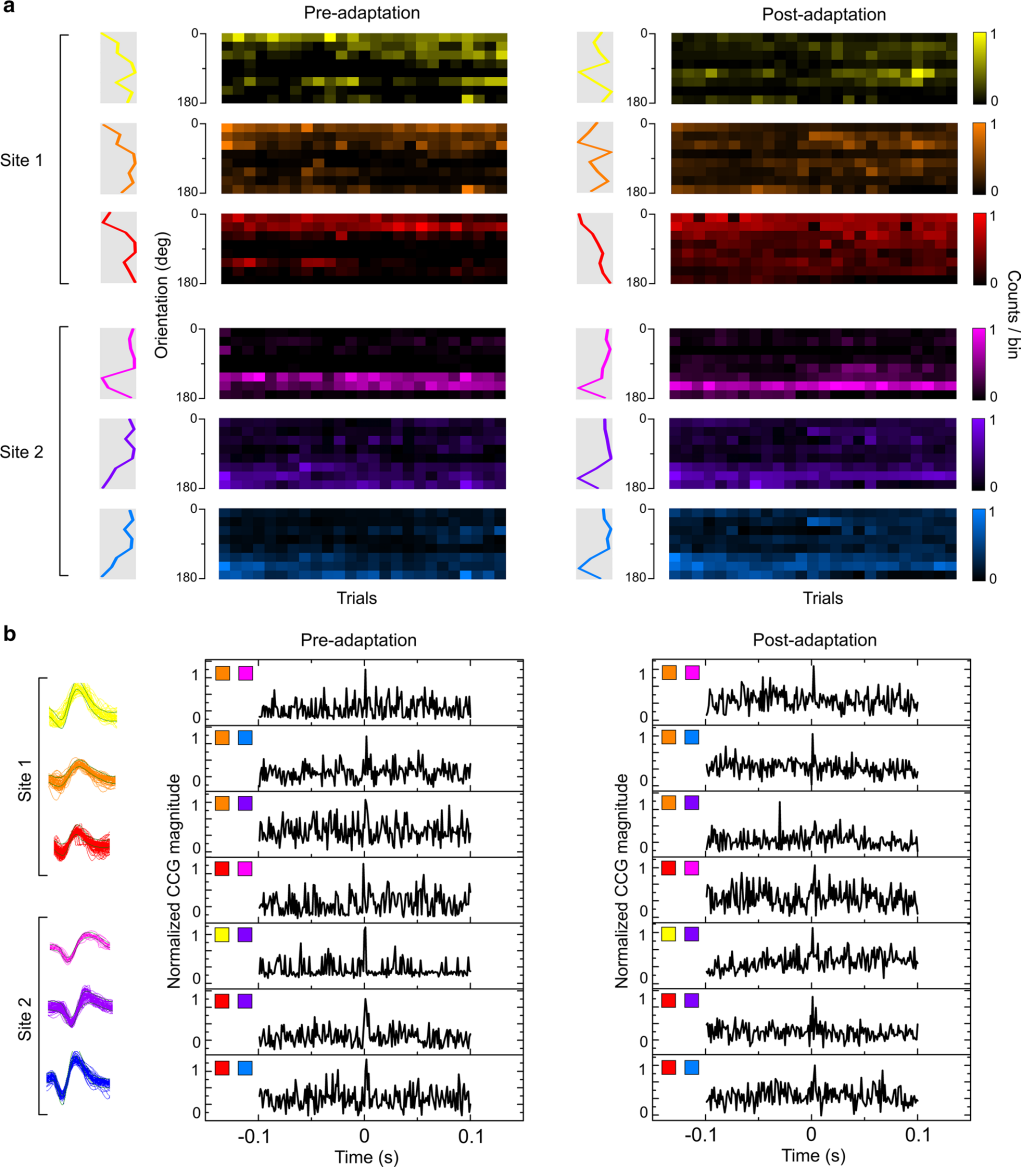
Modulation of functional connectivity. **a** Neuronal responses of six neurons recorded from two different sites. Colored matrices correspond to the measured responses at each trial (*X-axis*) and at all presented orientations (*Y-axis*). On the *left* are shown the raw tuning curves of each neuron. **b** Significant CCG’s between pairs of neurons from (**a**). The spike waveforms are displayed on the *left*

The previous results indicate that following adaptation, neurons modified their original orientation selectivity; hence the pixel-distribution of each orientation was reorganized. However the global distribution of orientations remained unmodified which is in line with previous report [23]. This raises one question: how the relationships between cells are modulated to maintain the equal distribution of orientations? Therefore, it becomes interesting to investigate how time-relationships between spikes of recorded neurons are changed. For this, we investigated the functional connectivity between neurons regardless of their orientation selectivity within sub-networks in pre- and post-adaptation phases. We recorded simultaneously the extracellular activity of groups of cells around two distinct electrodes (400 microns). Six examples of neuronal responses are shown in Fig. 3a (three cells from each site). Orientation tuning curves of all cells obtained from the raw data (response matrices) before and after adaptation are shown. Neuronal firing activity was crosscorrelated for all the possible neuron-pairs between the recorded sites. Based on the highest significant peaks across the CCGs of all pairs (pairs are coded by colored squares, the first colored square in the CCG represents the reference neuron, Fig. 3b, in this example all reference neurons were from site 1), putative functional connections were revealed within the networks and are further described in the next section. The spike waveforms of each neuron are displayed in Fig. 3b (left).

### Coordinated adjustment of synaptic weights in the circuits

**Fig. 4:**
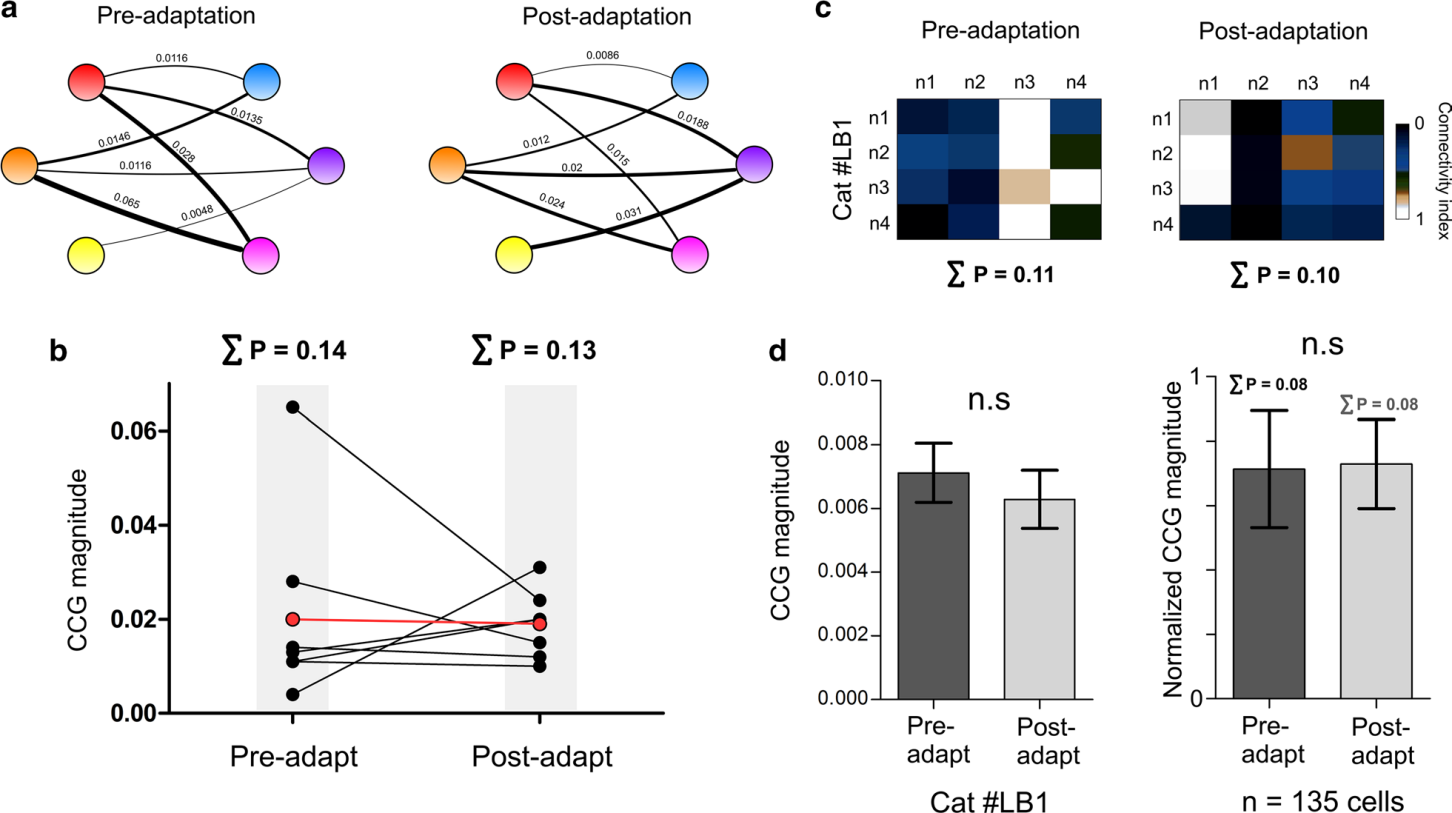
Connectivity dynamics of neuronal microcircuits. **a** Neuronal microcircuits and connectivity strength dynamics. The *thickness of lines* corresponds to the connectivity level. **b** Comparison between connectivity indices pre- and post-adaptation conditions. *Red dots* represent the mean connectivity strength. *Sums* are indicated above each condition. **c** Connectivity matrix of a different neuronal group showing the connectivity strength between all pairs. **d** Comparative histograms of the previous example (*left*) and global results (*right*)

We generated a connectivity circuit between the simultaneously recorded neurons shown previously (Fig. 4a). In the illustration, the value of CCG magnitude reflecting connection strength is indicated above each connecting-line, and is proportional to the thickness of the latter. The computations of CCG magnitudes of all summed pairs (n = 7) indicated a non-significant difference between the mean magnitude in pre- and post-adaptation conditions (paired two-tailed *t* test, p > 0.05, Fig. 4b). The sums (ΣP) of CCG magnitudes before and after visual adaptation were 0.14 and 0.13 respectively.

Another example is illustrated by a connectivity matrix (4 × 4 cells from two sites, Fig. 4c); it shows that the total connectivity strength remains unchanged following adaptation (the sums of CCG magnitudes were 0.11 and 0.10 before and after adaptation, respectively) despite the fact that functional connections are redeployed between different cells within the neuronal network (Fig. 4c). The connectivity matrices were found to be significantly different (Pearson coefficient = 0.11). Hence, there is an emergence of a new functional network within the same assembly that is characterized by the merging of both novel orientation selectivity’s and new links between cells wherein the functional strengths may weaken or strengthen. This suggests that there is a coordinated adjustment of synaptic strengths within the circuits leading to a rearrangement of functional connectivity with a change of orientation selectivity. However and most importantly, the overall proportion of connections is stable (histogram in Fig. 4d, left). The total values in all experiments pre- and post-adaptation point toward such stability (the summed CCG magnitude remained same at both pre and post-adaptation conditions, ΣP = 0.08, n = 135 cells, histogram in Fig. 4d, right).

### Interplay between selectivity and connectivity

**Fig. 5:**
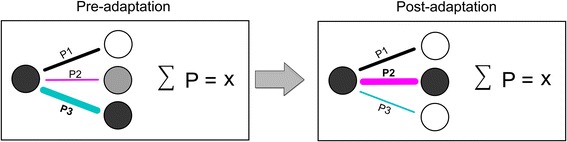
Mathematical model of connectivity pattern suggesting the stability of neuronal connectivity-strength after orientation-induced plasticity (*P1*, *P2* and *P3* represent the connection probabilities of each neuronal pair, *X* represents the total connectivity weight of the microcircuit)

Based on our findings, we propose a plastic neuronal network model which links the selectivity of neurons to their respective connectivity following plastic changes (Fig. 5). We found that the sum of the connectivity strength is equal at pre- and post-adaptation conditions. Mathematically, the total connectivity volume of a cell-assembly is the sum of all the individual contributions to the connectivity matrix; the connectivity strength is redistributed within the network following a “steady-selectivity-connectivity rule” so that the total connectivity volume of the entire assembly remains constant. This rule could be represented by the following equation:

$$ W = \mathop \sum \limits_{i = 1}^{n} CSi $$

$$ CS = \frac{N \times b}{t} $$

where *W* is the total connectivity weight within the network, *CS* is the measure of the individual Connectivity Strength for each cell-pair and *n* is the number of neuron-pairs, *t* represents the total time interval, *N* the number of spikes within this interval, and *b* represents the bin size of the calculated firing of the neuron (see “Methods”).

### Correlations as a function of adaptation-duration and distance between electrodes

**Fig. 6:**
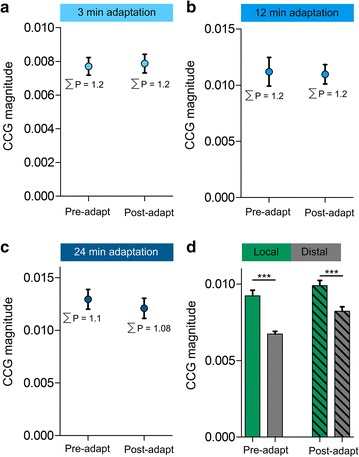
Correlations as a function of adaptation-duration [3 min in (**a**), 12 min in (**b**) and 24 min in (**c**)] and distance between neurons (**d**). The results indicate no significant difference in the summed connectivity-strengths between different adaptation-durations. However, significant differences were observed between locally recorded neurons (higher CCG magnitude) and neurons recorded from distinct electrode tips (lower CCG magnitude)

Finally, the effect of the time of adaptation on the connectivity-strength summation was investigated. For this purpose, three adaptation-durations were used: 3 min, 12 min and 24 min (Fig. 6a–c, respectively). The results indicate that the summed connectivity strengths are maintained post-adaptation with no significant effect of the time of adaptation. The results were: ΣP = 1.2 pre-adaptation and ΣP = 1.2 post-adaptation for both 3 and 12 min adaptation-duration, and ΣP = 1.1 pre-adaptation and ΣP = 1.08 for 24 min adaptation. Another parameter which has been tested is the distance between the recorded neurons. Multi-channel electrodes allowed simultaneously recording locally clustered cells as well as cells separated by up to 800 microns. The correlation-strength was then examined between neurons recorded from the same electrode tip (local, Fig. 6d) and between neurons recorded from distinct electrodes (distal, Fig. 6d). The results show significant differences between the connectivity strength of locally recorded neurons and the connectivity strength of distal neurons. These differences were observed for both pre- and post-adaptation phases (t-test, p < 0.01, Fig. 6d). Interestingly, for distal pairs, significant difference was also observed between pre- and post-adaptation phases with an increased average of the CCG magnitude (t-test, p < 0.01, Fig. 6d). This could be explained by an expansion of functional connections between neurons belonging to distinct microcircuits.

## Discussion

Sensory systems are continuously affected by the external environmental attributes [26]. The visual system is highly altered by experience wherein neuronal properties and neuronal circuits undergo important changes during development [6, 7]. These modifications may persist during adulthood as a consequence of large panoply of strategies such as visual deprivation [27, 28], retinal lesions [29] or adaptive sensory experience (short, long or repetitive visual exposure) [9–12, 30–32]. It appears that visual stimulation recruits functional groups of neurons which, when co-activated, process the visual stimuli properties [2]. Thus, the visual input from one microcircuit may affect the information provided to different downstream cell-assemblies [12, 26].

### Methodological considerations

From intrinsic optical imaging recordings, pixel-by-pixel subtraction computations allow the quantification of the difference between two orientation maps (in our case pre- and post-adaptation maps). It was thus important to test the stability of the maps as a control; indeed, the recorded signal may be contaminated by ambient noise due to breath-induced movement, light intensity change, cortical movement… etc. We have measured the light intensity of all generated images and found no difference between all animals as well as between all recording sessions. Despite the inherent presence of noise, the generated maps clearly contained well-organized regions with iso-orientation domains that converge at the center of pinwheels. This regular pattern cannot be attributed to random noise. In addition, orientation preference maps remained unchanged when tested twice (if no adaptation is applied) (Fig. 2a–c).

### The dynamic of neuronal connectivity

Brain processing is intimately related to how important the dynamic of complexly connected microcircuits is. Visual neurons within layers 2/3 are selectively interconnected leading to the emergence of independent fine-scale circuits entrenched in the cortical architecture [1]. In line with our data, experience-dependent plasticity leads to modifications of the connectivity patterns within the neuronal network. However, it has been proposed that a homeostatic process is established in order to stabilize the global connectivity strength of the neuronal group [19]. This regulating activity is considered as a complementary process to the Hebbian plasticity where changes in synaptic strengths are observed after plasticity in order to refine the properties of neuronal-assemblies [19–21]. In young animals for instance, stimulus adaptation leads to the development of orientation maps. Exposing kittens to a single oriented environment shifted the optimal orientations of many neurons to the experienced orientation [7]. Hence, experience is a significant factor in determining the plastic changes operating in orientation maps [7]. In adulthood, there have been studies reporting the adapting ability of the visual cortex to external stimuli: orientation, contrast, motion, direction and spatial frequency [9–11, 30, 31, 33]. Stimulus exposure (adaptation) which mimics the learning process changes neuronal properties; this may be attributed to changes in dynamics of neuronal cell-assemblies wherein neurons acquire new optimal properties [8, 11, 32]. Recent findings assign a high contribution of adaptation to neuronal response uniformity within a population of cells sharing neuronal and feature selectivity [5, 34]. Moreover, it has been shown that adaptation enhances the spike-synchrony in the gamma frequency range in V4 [35] as well as in V1 [36]. This gamma-modulation of synchrony is coupled with an improvement of feature encoding [35, 36].

Functional cell-assemblies are newly formed wherein neurons sharing similar stimulus preference exhibit high connectivity profile [4, 37]. On the other hand, during the critical period and learning in adulthood, synaptic strengths have to be modified in order to regulate the neuronal properties changes due to multiple synaptic drives and maintain a stable level of firing [17]. In line with our findings, homeostatic scaling was proposed as a strategy to normalize the global synaptic connectivity strength to compensate the Hebbian plasticity which modifies the inter-cellular connectivity in relation to the neuronal selectivity [38]. Homeostatic plasticity prevents an over-increase or over-decrease of the firing activity levels due to long-term potentiation (LTP) or long-term depression (LTD) which modify the connectivity strength in order to change the neuronal selectivity features [39–41]. Another scenario would be the change in excitation-inhibition equilibrium that could lead to critical effects on neuronal spiking activity and information processing [18, 42]. Maintaining stable excitation-inhibition ratio could thus prevent an exceeding augmentation or diminution of global strengths within networks allowing sensory processing to remain stable [18].

## Conclusion

The concept of neuronal homeostasis implies that synaptic weights could be limited to an optimal level in order to regulate the total connectivity ratio within the assemblies. Indeed, the increase of synaptic strengths within a group of neurons may lead to the decrease of other connections as a trade-off allowing, therefore, the formation of new functional microcircuits after plasticity.

## Abbreviations

OSI: orientation selectivity index
CS: connectivity strength
RF: receptive field
CCG: crosscorrelogram
